# Effect of optical diagnosis training on recognition and treatment of submucosal invasive colorectal cancer in community hospitals: a prospective multicenter intervention study

**DOI:** 10.1055/a-2313-4996

**Published:** 2024-06-11

**Authors:** Lonne W.T. Meulen, Krijn J.C. Haasnoot, Marije S. Vlug, Frank H.J. Wolfhagen, Martine A.M.C. Baven-Pronk, Michael P.J.A. van der Voorn, Matthijs P. Schwartz, Lauran Vogelaar, Wouter H de Vos tot Nederveen Cappel, Tom C.J. Seerden, Wouter L. Hazen, Ruud W.M. Schrauwen, Lorenza Alvarez-Herrero, Ramon-Michel Schreuder, Annick B. van Nunen, Esther Stoop, Gijs J. de Bruin, Philip Bos, Willem A. Marsman, Edith Kuiper, Marc de Bièvre, Yasser A. Alderlieste, Robert Roomer, John Groen, Marloes Bigirwamungu-Bargeman, Peter D. Siersema, Sjoerd G. Elias, Ad A.M. Masclee, Leon M.G. Moons, on behalf of the OPTICAL-STAR study team and the Dutch T1 CRC Working Group

**Affiliations:** 1 Department of Gastroenterology and Hepatology, Maastricht University Medical Center, Maastricht, The Netherlands; 2 GROW, School for Oncology and Reproduction, Maastricht University, Maastricht, The Netherlands; 3 Department of Gastroenterology and Hepatology, University Medical Centre Utrecht, Utrecht, The Netherlands; 4 Department of Gastroenterology and Hepatology, Dijklander Hospital, Hoorn, The Netherlands; 5 Department of Gastroenterology and Hepatology, Albert Schweitzer Hospital, Dordrecht, The Netherlands; 6 Department of Gastroenterology and Hepatology, Groene Hart Hospital, Gouda, The Netherlands; 7 Department of Gastroenterology and Hepatology, Haga Hospital, Den Haag, The Netherlands; 8 Department of Gastroenterology and Hepatology, Meander Medical Center, Amersfoort, The Netherlands; 9 Department of Gastroenterology and Hepatology, Diakonessenhuis, Utrecht, The Netherlands; 10 Department of Gastroenterology and Hepatology, Isala Clinics, Zwolle, The Netherlands; 11 Department of Gastroenterology and Hepatology, Amphia Hospital, Breda, The Netherlands; 12 Department of Gastroenterology and Hepatology, Elisabeth-Tweesteden Hospital, Tilburg, The Netherlands; 13 Department of Gastroenterology and Hepatology, Bernhoven, Uden, The Netherlands; 14 Department of Gastroenterology and Hepatology, Sint Antonius Hospital, Nieuwegein, The Netherlands; 15 Department of Gastroenterology and Hepatology, Catharina Hospital Eindhoven, Eindhoven, The Netherlands; 16 Department of Gastroenterology and Hepatology, Zuyderland Medical Center, Sittard-Geleen, The Netherlands; 17 Department of Gastroenterology and Hepatology, Haaglanden Medical Center, Den Haag, The Netherlands; 18 Department of Gastroenterology and Hepatology, Tergooi Hospital, Hilversum, The Netherlands; 19 Department of Gastroenterology and Hepatology, Gelderse Vallei Hospital, Ede, The Netherlands; 20 Department of Gastroenterology and Hepatology, Spaarne Gasthuis, Haarlem, The Netherlands; 21 Department of Gastroenterology and Hepatology, Maasstad Hospital, Rotterdam, The Netherlands; 22 Department of Gastroenterology and Hepatology, Viecuri Medical Center, Venlo, The Netherlands; 23 Department of Gastroenterology and Hepatology, Rivas, Gorinchem, The Netherlands; 24 Department of Gastroenterology and Hepatology, Franciscus Gasthuis, Rotterdam, The Netherlands; 25 Department of Gastroenterology and Hepatology, Sint Jansdal Hospital, Harderwijk, The Netherlands; 26 Department of Gastroenterology and Hepatology, Medical Spectrum Twente, Enschede, The Netherlands; 27 Department of Gastroenterology and Hepatology, Radboud University Medical Center, Nijmegen, The Netherlands; 28 Julius Center for Health Sciences and Primary Care, University Medical Center Utrecht, Utrecht, the Netherlands

## Abstract

**Background** Recognition of submucosal invasive colorectal cancer (T1 CRC) is difficult, with sensitivities of 35 %–60 % in Western countries. We evaluated the real-life effects of training in the OPTICAL model, a recently developed structured and validated prediction model, in Dutch community hospitals.

**Methods** In this prospective multicenter study (OPTICAL II), 383 endoscopists from 40 hospitals were invited to follow an e-learning program on the OPTICAL model, to increase sensitivity in detecting T1 CRC in nonpedunculated polyps. Real-life recognition of T1 CRC was then evaluated in 25 hospitals. Endoscopic and pathologic reports of T1 CRCs detected during the next year were collected retrospectively, with endoscopists unaware of this evaluation. Sensitivity for T1 CRC recognition, R0 resection rate, and treatment modality were compared for trained vs. untrained endoscopists.

**Results** 1 year after e-learning, 528 nonpedunculated T1 CRCs were recorded for endoscopies performed by 251 endoscopists (118 [47 %] trained). Median T1 CRC size was 20 mm. Lesions were mainly located in the distal colorectum (66 %). Trained endoscopists recognized T1 CRCs more frequently than untrained endoscopists (sensitivity 74 % vs. 62 %; mixed model analysis odds ratio [OR] 2.90, 95 %CI 1.54–5.45). R0 resection rate was higher for T1 CRCs detected by trained endoscopists (69 % vs. 56 %; OR 1.73, 95 %CI 1.03–2.91).

**Conclusion** Training in optical recognition of T1 CRCs in community hospitals was associated with increased recognition of T1 CRCs, leading to higher en bloc and R0 resection rates. This may be an important step toward more organ-preserving strategies.

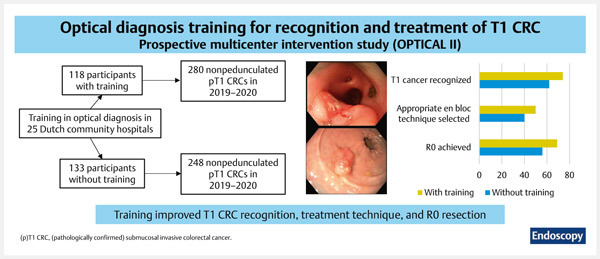

## Introduction

Adequate recognition of submucosal invasive colorectal cancer (T1 CRC) in nonpedunculated colorectal polyps is essential in selecting polyps for an adequate local resection technique that aims to achieve R0 resection 1 . Unfortunately, optical diagnosis of T1 CRCs is still challenging, with sensitivities ranging between 35 % and 60 % 2 3 4 5 . Therefore, a proportion of T1 CRCs are not recognized until histological assessment, causing difficulties in risk stratification due to fragmentation and improper orientation of the specimen.

**Fig. 1:**
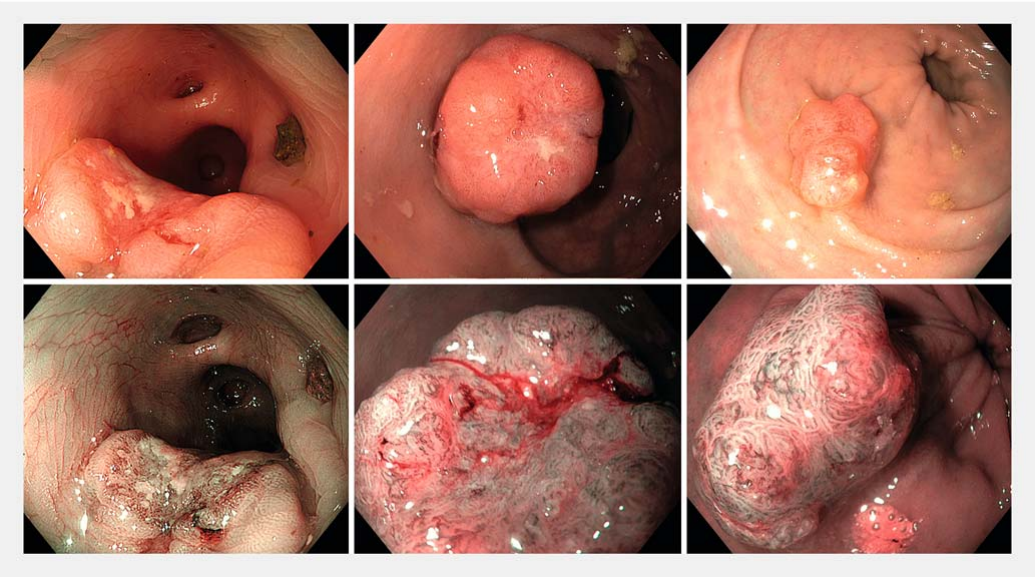
Three submucosal invasive colorectal cancers in white-light and advanced imaging.

While enhanced imaging with either zoom chromoendoscopy, narrow-band imaging, or blue-light imaging is essential for the correct diagnosis of T1 CRCs 6 7 , white-light features, such as size, location, and surface morphology, are also helpful in stratifying polyps into high and low risk lesions 8 9 10 ( Fig. 1 ). Models incorporating both enhanced imaging features as well as morphological features have recently been reported 5 11 12 . In the Netherlands, the validated OPTICAL model was developed, discriminating T1 CRCs and noninvasive, nonpedunculated polyps of ≥ 20 mm with a sensitivity and specificity of 78.7 % and 94.2 %, respectively 12 . This model supports endoscopists in applying dedicated local excision techniques for high risk lesions. However, most of these models have been validated using images, and have included only patients already selected for endoscopic submucosal dissection (ESD) or endoscopists who were interested in polyp characterization 11 13 14 15 16 17 18 . It is also unknown whether implementing such a model improves clinical outcomes, such as increased T1 CRC recognition, higher proportions of R0 resection, and whether it will decrease surgery rates after its implementation in community hospitals.

In this multicenter prospective study (OPTICAL II), we evaluated whether training in the OPTICAL model in community hospitals led to better treatment outcomes for patients with T1 CRC, by comparing outcomes (R0 resection, en bloc resection, and treatment strategy) between trained and untrained endoscopists.

## Methods

### Study design and source population

**Fig. 2:**
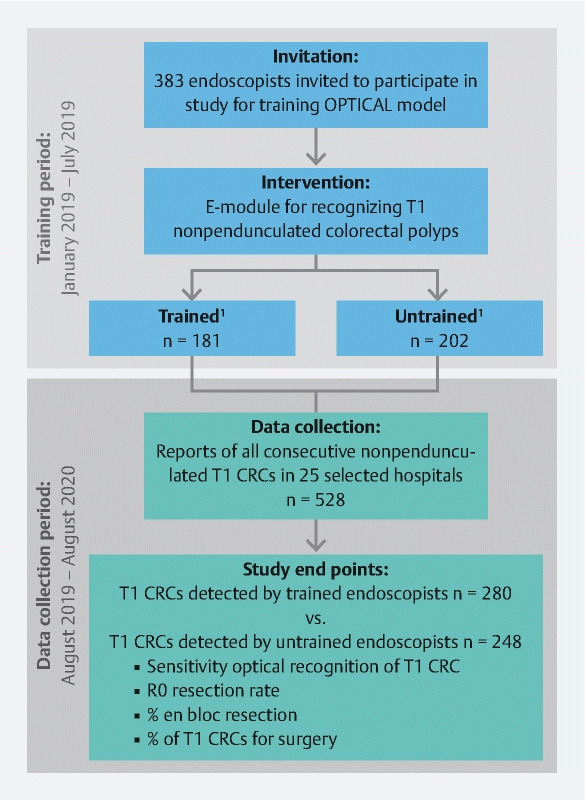
Flow chart of study design and inclusions. ^1^ Trained endoscopists completed the e-learning program (with or without post-test). Untrained endoscopists did not register or dropped out during the e-learning program. T1 CRC, submucosal invasive colorectal cancer.

This was a prospective multicenter study from the Dutch T1 CRC Working Group, conducted from January 2019 to August 2020. All 383 endoscopists from 40 Dutch hospitals were invited to participate in the study ( Fig. 2 ) and were granted access to voluntarily pass through an e-learning program explaining the features of the OPTICAL model 12 . In short, the e-learning program consisted of 40 practice cases, an e-course displaying online movies explaining the features of the OPTICAL model, and an explanation of various example cases. The e-learning program trained endoscopists in the recognition of T1 CRCs, but also in the selection of cases for a “dedicated en bloc resection technique” defined as ESD, endoscopic intermuscular dissection (EID), endoscopic full-thickness resection (eFTR), transanal endoscopic microsurgery (TEM), transanal minimally invasive surgery (TAMIS), or combined endoscopic laparoscopic surgery (CELS). Details of the e-learning can be found in **Supplementary material Part 1s** , **Table 1s,** and **Fig. 1s** in the online-only Supplementary material.

A trained endoscopist was defined as an endoscopist who completed the preceding practice cases and e-learning program ( **Fig. 2s** ), without the necessary completion of the final practice cases (trained; n = 181). We considered endoscopists untrained if they did not participate in the e-learning program, or only performed the preceding practice cases (untrained; n = 202). Among the trained participants were 136 gastroenterologists, 24 nurse endoscopists, and 21 residents in training. Access to the e-learning program was granted until July 2019.

### Real-life clinical practice

To evaluate the effect of the e-learning program, we collected all pathology reports of T1 CRCs diagnosed after the training period, from August 2019 until August 2020, in a random selection of 25 hospitals with varying levels of participation in the e-learning program. All consecutive pathologically confirmed T1 CRCs during this period were identified by performing a search in the nationwide registry of histo- and cytopathology in the Netherlands 19 . To prevent bias, participants were unaware of the inclusion of their encountered T1 CRCs, and as such, they were blinded to this part of the study. Data were collected retrospectively, but as we collected all pathologically confirmed T1 CRCs, we could include all consecutive T1 CRC lesions encountered by the participating endoscopists irrespective of treatment modality.

We evaluated whether the endoscopist had recognized the lesion as at risk for T1 CRC by analyzing the corresponding endoscopy report. There were no standardized report forms, so any statement in the endoscopy report leading to suspicion of submucosal invasion (e. g. suspicious for T1 CRC, Narrow-band imaging International Colorectal Endoscopic classification 3, Kudo V, Hiroshima C2-C3, or uncertainty about noninvasiveness) was registered as an adequate recognition of T1 CRC. We chose this approach because we expected that the suspicion of submucosal invasion would lead to the selection of the same resection techniques as those that would be selected for a lesion with T1 CRC. One reader extracted all data, and a second reader checked a representative sample of 5/25 centers, corresponding to 16 % of all cases. Cases with discrepancies between the two readers were discussed with the principal investigator of the study.

Next, we collected data on the treatment performed (endoscopic, primary, or secondary surgery) and outcome (negative resection margins [R0] and en bloc resection). In our cohort, three options were observed in the course of optical diagnosis toward treatment: (1) T1 CRC recognized, biopsies, and referral for primary surgery; (2) recognized T1 CRC, primary local excision performed; (3) uncertainty in optical diagnosis of T1 CRC, biopsies, and subsequent decision on treatment strategy (local excision or surgery). Finally, we compared these outcomes between trained and untrained endoscopists. To correct for a possible selection bias toward already more dedicated endoscopists that finished the e-learning, we obtained additional information on endoscopists’ characteristics. The principal investigator of each participating center provided details of the following characteristics for both trained and untrained endoscopists: prior training in optical diagnosis, certification to participate in the Dutch population-based CRC screening program, endoscopy experience in years, focus of expertise, use of (virtual) chromoendoscopy, use of endoscopic mucosal resection (EMR) for lesions ≥ 20 mm, use of advanced endoscopic techniques (ESD, eFTR, EID), total colonoscopies per year, dedication in recognition of T1 CRCs, and frequency of consulting an expert colleague during colonoscopy ( **Table 2s** ). Morphology was defined as “sessile or sessile component” versus “flat” (when no sessile component was described). Morphology was not defined according to the Paris classification or that for laterally spreading tumors because these are not registered in a standardized manner in the Netherlands.

A directed acyclic graph was made to provide insight into parameters influencing T1 CRC recognition and showing which parameters were available in our cohort ( **Fig. 3s** ).

The Medical Ethical Review Committee of the Maastricht University Medical Center approved the study and waived the need for informed consent (2021–2719). Patients or the public were not involved in the design, conduct, or reporting of this research. We used the Standards for Reporting Diagnostic accuracy studies checklist when writing our report and followed the Sex and Gender Equity in Research guidelines for sex and gender reporting 20 21 .

### Statistical analysis

For descriptive statistics, categorical variables are presented as number and percentage, and numerical variables are presented as median and interquartile range (IQR) or mean and SD. Pearson’s chi-squared method and Fisher’s exact test were used to assess differences in baseline patient, lesion, and endoscopist characteristics between groups. Generalized linear mixed models with logit link were used to assess differences in (binary) outcomes between T1 CRCs detected by trained vs. untrained endoscopists. A random intercept on endoscopist level was included to account for the correlation between T1 CRCs assessed by the same endoscopist. A generalized linear mixed model with logit link and random intercept on endoscopist level was also used to evaluate the independent effect of training on T1 CRC sensitivity, with correction for observed associations of size, morphology, and location of the lesion, the indication of colonoscopy, and endoscopists’ experience or dedication to the treatment of colorectal polyps. The directed acyclic graph shows for which parameters correction was not possible ( **Fig. 3s** ). Sensitivity analysis was performed, in which an additional random intercept on treatment center was included to account for the correlation between T1 CRCs assessed by endoscopists from the same treatment center. Additional sensitivity analysis was performed, in which the intervention was excluded from the model, to investigate whether there was high interaction in the model.

R version 3.5.1 (R Foundation for Statistical Computing, Vienna, Austria) and SPSS version 27.0.0 (IBM Corp, Armonk, New York, USA) were used for statistical analysis and figures.

## Results

### Optical e-learning

Of 383 invited endoscopists, 181 (47 %) participants completed the e-learning program. In this training structure, overall diagnostic accuracy for recognizing T1 CRC (superficially or deeply invasive) vs. adenoma (low grade or high grade dysplasia) was high in the online e-learning environment, as demonstrated by the results of the practice cases. In the preceding practice cases, a sensitivity of 82 % (95 %CI 80 %–84 %), specificity of 72 % (95 %CI 71 %–74 %), positive predictive value of 50 % (95 %CI 48 %–52 %), and negative predictive value of 93 % (95 %CI 92 %–93 %) were observed. In the final practice cases, a sensitivity of 82 % (95 %CI 80 %–84 %), specificity of 73 % (95 %CI 71 %–74 %), positive predictive value of 50 % (95 %CI 48 %–52 %), and negative predictive value of 92 % (95 %CI 92 %–93 %) were observed.

Endoscopists scoring high sensitivities (80 %–100 %) in this training structure were not more experienced than low performers (< 80 % sensitivity), and were not more dedicated to recognition of T1 CRCs, or certified for the bowel cancer screening program, or performing advanced resection techniques more often than low performers.

Further details of the e-learning and practice cases can be found in the **Supplementary material Part 1s** .

### Real-life clinical practice

#### Study population and included T1 CRCs

In 25 hospitals, we included all consecutively detected T1 CRCs for 1 year after the training period of 118 trained and 133 untrained endoscopists, resulting in a total of 660 T1 CRCs, of which 528 (80 %) were nonpedunculated. A total of 280 (53 %) of these nonpedunculated T1 CRCs were from colonoscopies performed by endoscopists who were trained in the OPTICAL model, while 248 (47 %) were from colonoscopies performed by untrained endoscopists. Characteristics of patients and included T1 CRCs are presented in Table 1 , and the characteristics of trained vs. untrained endoscopists are presented in **Table 2s** .

**Table 1:** Patient and lesion characteristics of pathologically confirmed nonpedunculated submucosal invasive (T1) colorectal cancers from colonoscopies performed by endoscopists with and without training in optical diagnosis.

	Overall T1 CRCs (N = 528)	T1 CRCs from colonoscopies performed by trained endoscopists (N = 280)	T1 CRCs from colonoscopies performed by untrained endoscopists (N = 248)	*P* value
**Patient characteristics**				
Female sex, n (%)	238 (45)	116 (41)	122 (49)	0.07
Age, mean (SD), years	69 (9.1)	68 (9.5)	70 (8.7)	0.15
ASA classification, n (%)				0.74
I	101 (19)	53 (19)	48 (19)	
II	354 (67)	184 (66)	170 (69)	
III	71 (13)	42 (15)	29 (12)	
IV	2 (1)	1 (0)	1 (0)	
**Lesion characteristics**				
Size, median (IQR), mm	20 (15)	20 (15)	20 (15)	0.86
Size groups, n (%)				0.60
1–5 mm	6 (1)	5 (2)	1 (0)	
6–9 mm	26 (5)	16 (6)	10 (4)	
10–19 mm	174 (33)	88 (31)	86 (35)	
20–29 mm	170 (32)	91 (33)	79 (32)	
30–39 mm	79 (15)	42 (15)	37 (15)	
≥ 40 mm	73 (14)	38 (14)	35 (14)	
Morphology, n (%)				0.048
Sessile or sessile component	341 (65)	193 (69)	149 (60)	
Flat	187 (35)	87 (31)	99 (40)	
Location, n (%)				0.31
Proximal 1	182 (34)	91 (33)	91 (37)	
Distal	346 (66)	189 (67)	157 (63)	
Location rectum, n (%)	214 (41)	111 (40)	103 (42)	0.66
Indication colonoscopy, n (%)				0.04
Screening	252 (48)	131 (47)	121 (49)	
Surveillance	42 (8)	22 (8)	20 (8)	
Diagnostic	195 (37)	99 (35)	96 (39)	
Therapeutic	29 (5)	23 (8)	6 (2)	
Missing	10 (2)	5 (2)	5 (2)	
Treatment, n (%)				0.49
Local excision	234 (44)	127 (45)	107 (43)	
Primary surgery	211 (40)	114 (41)	97 (39)	
Secondary surgery 2	83 (16)	39 (14)	44 (18)	

CRC, colorectal cancer; ASA, American Society of Anesthesiologists; IQR, interquartile range.

1 Proximal location is defined as cecum, ascending colon, and tranversum including the splenic flexure.

2 Secondary surgery is defined as surgery following a local excision of a T1 CRC.

T1 CRCs were equally distributed between male and female patients (45 % female). Registration of all individual OPTICAL parameters in the endoscopy reports was completed for 58/280 (21 %) T1 CRCs detected by trained endoscopists, compared with 7/248 (3 %) T1 CRCs detected by untrained endoscopists.

#### Recognition of T1 CRCs

Primary outcomes are presented in Table 2 . Trained endoscopists showed better recognition of nonpedunculated T1 CRCs compared with untrained endoscopists (74 % vs. 62 %, *P* = 0.006; OR 1.74, 95 %CI 1.18–2.56). No clinically relevant differences in baseline cancer characteristics were observed between T1 CRCs detected by trained endoscopists vs. untrained endoscopists ( Table 1 ). Trained endoscopists were more experienced in EMR and (virtual) chromoendoscopy, performed advanced resection techniques more often (EMR for lesions > 20 mm, ESD, or eFTR), and were certified for the screening program more frequently than untrained endoscopists ( **Table 2s** ). To correct for potential selection bias of pre-existing levels of training and dedication, multivariable regression analysis was performed, correcting for both lesion characteristics (colonoscopy indication, size, morphology, and location of the lesion) and endoscopist characteristics (experience, focus of expertise, screening program certification, and dedication to lesion characterization) ( Table 3 ). This showed that training in the OPTICAL model remained significantly associated with a higher sensitivity for T1 CRCs in clinical practice (OR 2.90, 95 %CI 1.54–5.45). Other independent factors influencing sensitivity for T1 CRCs were lesion size and morphology. Sessile morphology was associated with lower sensitivity for T1 CRCs compared with flat morphology (56 % vs. 81 %; OR 0.29, 95 %CI 0.16–0.52). Lesion size of < 20 mm or > 40 mm was associated with lower sensitivity for T1 CRC compared with a lesion size of 20–40 mm (61 % in < 20 mm lesions; OR 0.44, 95 %CI 0.26–0.76; 78 % in 20–40 mm lesions; 58 % in > 40 mm lesions; OR 0.26, 95 %CI 0.12–0.58). Recognition of T1 CRCs was similar for endoscopists who reported the OPTICAL parameters in their endoscopy reports, compared with endoscopists who did not (76 % vs. 73 %; OR 1.05, 95 %CI 0.56–1.97; *P* = 0.88). Sensitivity analysis including treatment center showed similar outcomes. Sensitivity analysis excluding the intervention also showed similar outcomes for other variables in the model, indicating absence of an interaction effect.

**Table 2:** Diagnosis and treatment outcomes of nonpedunculated submucosal invasive (T1) colorectal cancers.

	Overall T1 CRCs (N = 528), n/N (%)	ICC 1	T1 CRCs from colonoscopies performed by trained endoscopists (N = 280), n/N (%)	T1 CRCs from colonoscopies performed by untrained endoscopists (N = 248), n/N (%)	OR (95 %CI) 2	*P* value
Recognition (sensitivity), n (%)	361 /528 (68)	0.06	207 /280 (74)	154 /248 (62)	1.74 (1.18–2.56)	0.006
Proportion of en bloc resections (local excised T1 CRCs)	239 /317 (75)	0.08	128 /166 (77)	111 /151 (74)	0.82 (0.48–1.41)	0.47
Dedicated local en bloc resection technique used 3	143 /317 (45)	0.19	83 /166 (50)	60 /151 (40)	1.59 (0.95–2.67)	0.08
R0 resection rate (local excised T1 CRCs)	200 /317 (63)	0.16	115 /166 (69)	85 /151 (56)	1.73 (1.03–2.91)	0.04
Proportion of primary surgery	211 /528 (40)	0.10	114 /280 (41)	97 /248 (39)	1.11 (0.76–1.62)	0.60

CRC, colorectal carcinoma; ICC, intraclass coefficient; OR, odds ratio.

1 Intraclass coefficient reflects the correlation between the outcomes of patients within the same endoscopist.

2 Generalized linear mixed model with a random intercept on endoscopist to correct for clustering.

3 Dedicated local en bloc resection technique defined as: endoscopic submucosal dissection, endoscopic intermuscular dissection, endoscopic full-thickness resection, transanal endoscopic microsurgery, transanal minimally invasive surgery, or combined endoscopic laparoscopic surgery.

**Table 3:** Mixed model analysis on recognition of submucosal invasive (T1) colorectal cancers.

	Coefficient	SE	*P* value 1	OR	95 %CI
Trained in OPTICAL model	1.064	0.321	0.001	2.90	1.54–5.45
Size of lesion					
< 20 mm	–0.816	0.274	0.003	0.44	0.26–0.76
20–40 mm	Ref				
> 40 mm	–1.347	0.409	0.001	0.26	0.12–0.58
Location, rectum	0.049	0.269	0.86	1.05	0.62–1.78
Indication for colonoscopy					
Screening	Ref				
Surveillance	0.345	0.461	0.45	1.41	0.57–3.49
Diagnostic (symptomatic)	0.404	0.313	0.20	1.50	0.81–2.77
Therapeutic (referred)	–0.280	0.567	0.62	0.76	0.25–2.30
Diagnostic (other)	0.501	0.575	0.38	1.65	0.53–5.12
Morphology – sessile/sessile component	–1.241	0.295	< 0.001	0.29	0.16–0.52
BBPS score ≥ 2 per segment 2	0	–	–	–	–
Endoscopist factors					
Screening program certified	–0.332	0.422	0.43	0.72	0.31–1.65
Perform EMR ≥ 20 mm	–0.496	0.402	0.21	0.61	0.38–1.34
Use advanced endoscopy technique	0.089	0.419	0.83	1.09	0.48–2.49
Focus area – colorectal	–0.210	0.387	0.59	0.81	0.38–1.73
Use (virtual) chromoendoscopy	–0.426	0.392	0.28	0.65	0.30–1.41
Dedicated T1 CRC recognition	0.503	0.426	0.24	1.65	0.72–3.82
Frequently consulting expert	0.263	0.465	0.58	1.30	0.52–3.25
Experience in years					
0–5 years	Ref				
6–10 years	–0.535	0.410	0.19	0.59	0.26–1.31
> 10 years	–0.314	0.399	0.43	0.73	0.33–1.60
Intercept	1.975	0.772	0.01		

OR, odds ratio; BBPS, Boston Bowel Preparation Scale; EMR, endoscopic mucosal resection; CRC, colorectal carcinoma.

1 Generalized linear mixed models (with logit link), with random intercept on endoscopist level.

2 BBPS score was ≥ 2 for all cases.

#### Local excision outcomes for T1 CRCs

Although no differences existed in the proportion of en bloc resections, trained endoscopists more often selected a dedicated en bloc resection technique for recognized T1 CRCs (50 % vs. 40 %; OR 1.59, 95 %CI 0.95–2.67; *P* = 0.08) ( Table 2 ). Furthermore, the R0 resection rate after local excision of T1 CRCs was higher in trained vs. untrained endoscopists (69 % vs. 56 %; OR 1.73, 95 %CI 1.03–2.91; *P* = 0.04).

With increasing size of the lesion, the en bloc resection rate decreased (84 % in < 20 mm, 79 % in 20–40 mm, and 56 % in > 40 mm), and the primary surgery referral percentage increased (22 % in < 20 mm, 54 % in 20–40 mm, and 44 % in > 40 mm).

#### Variability of outcomes between participating centers

To assess whether uptake of the e-learning program per center (i. e. the percentage of all endoscopists in a specific center who finished the program) influenced T1 CRC diagnosis and treatment outcomes, participating centers were compared regarding the outcomes ( **Fig. 4s, Fig. 5s** ). While a variation between centers was seen for T1 CRC recognition (mean 68 % [SD 46.5], range 29 %–91 %), proportion of R0 resection (mean 63 % [SD 24.0], range 0 %–100 %), and proportion of primary surgery (mean 43 % [SD 16.8], range 11 %–75 %), these differences were not observed when centers were categorized according to the percentage of endoscopists participating in the OPTICAL e-learning (< 30 % of the centers’ endoscopists vs. 30 %–70 % vs. ≥ 70 %). In mixed model analysis, the difference in T1 CRC recognition between centers appeared to be caused by endoscopist and polyp characteristics. The addition of treatment center did not lead to a better discrimination (random effect 0.000).

## Discussion

In this prospective, multicenter study, we evaluated the effects of nationwide training in optical diagnosis on the sensitivity for detecting T1 CRCs. Training was shown to be an independent predictor for better optical recognition of nonpedunculated T1 CRCs (OR 2.90, 95 %CI 1.54–5.45). Furthermore, this study showed that the recognition of T1 CRCs led to a better treatment strategy, reflected by more frequent use of dedicated local excision techniques, and a higher percentage of R0 resections of T1 CRCs identified by trained endoscopists (69 % vs. 56 %; OR 1.73, 95 %CI 1.03–2.91; *P* = 0.04).

An R0 resection not only optimizes histological risk stratification and identification of a group at low risk of lymph node metastasis, but it can also be the starting point of an organ-preserving treatment strategy even in the presence ≥ 1 risk factor 22 . Optimizing the chance of an R0 resection by selecting the most appropriate resection technique should be the major aim of pre-resection assessment. In our cohort, however, EMR was still frequently chosen as a resection technique for small T1 CRCs, despite lesions being recognized as early cancer. The R0 resection rate of only 46 % after treatment of a T1 CRC with EMR irrespective of size is in line with a previously reported R0 resection rate of 59 % after intentional EMR for T1 CRCs 23 . The R0 resection potential of EMR is likely being overestimated in smaller-sized T1 CRCs. Consequently, EMR should be discouraged as a first-line treatment for suspected T1 CRCs.

The difference in R0 resection rates for detected T1 CRCs between trained and untrained endoscopists was more prominent in colonic than rectal T1 CRCs. This was partly due to more frequent selection of an en bloc resection technique for large polyps in the rectum independently of the recognition of T1 CRCs.

In contrast to Vleugels et al. 2 , who observed that recognized T1 CRCs were less frequently referred for surgery, increased recognition did not result in a decrease of surgery in our study (data not shown). Although the suspected depth of invasion was not registered in the endoscopy report, more obvious signs of cancer, and therefore increased risk of deeper submucosal invasion, may have been the main reason for referral to surgery. However, depth of submucosal invasion has recently been recognized as a weak predictor of lymph node metastasis, with an absolute risk of only 2.6 % when deep submucosal invasion is the sole risk factor present 24 . The use of en bloc resection techniques, such as eFTR 25 , TAMIS, CELS 26 , or EID 27 , instead of primary surgery, should therefore be considered as the first approach for removal of deeply invasive T1 CRCs. By performing en bloc resections for all T1 CRCs, including those with deep submucosal invasion, another subgroup may be recognized as eligible for intensive follow-up or adjuvant chemoradiotherapy.

Several limitations of our study should be acknowledged. The selection of more dedicated endoscopists may have occurred in the trained group. Given the voluntary nature of study participation, there might be a bias toward endoscopists already dedicated to T1 CRC recognition. Comparing trained and untrained endoscopists’ characteristics showed more dedication to T1 CRC recognition, CRC screening program certification, and more use of advanced endoscopy techniques among trained endoscopists. However, after correction for these differences, training in the OPTICAL model remained an independent predictor for T1 CRC recognition. Therefore, we believe that although dedicated endoscopists were more likely to complete the training, the training still led to improved T1 CRC recognition, and, more importantly, a better treatment strategy. Furthermore, the directed acyclic graph ( **Fig. 3s** ) showed some unmeasured confounders that we could not correct for in our analysis. However, we believe the influence of these unmeasured confounders is limited, as these parameters are related to other parameters (e. g. number of screening colonoscopies is related to years of experience and screening certification) or very unlikely to be different between groups (e. g. time pressure), or might be a mediator instead of a confounder (e. g. sufficient cleaning of the polyp). Thus, while a part of the effect might be explained by these unmeasured differences between endoscopists or procedural differences, it is highly unlikely that the complete training effect might be explained by unmeasured confounders.

The sensitivity for detecting T1 CRCs in clinical practice in our study (68 % overall, 74 % for trained endoscopists, and 62 % for untrained endoscopists) is high compared with reported sensitivities of 35 %–60 % in previous cohort studies, especially for untrained endoscopists 2 3 4 5 . This high sensitivity of untrained endoscopists for detecting T1 CRC may be explained by several factors. First, some studies were performed in the early years after implementation of the CRC screening program (2015–2017) 2 3 5 . Given the 6 years of experience within the CRC screening program at the time of our study, we might be observing a natural learning curve for T1 CRC recognition. Second, due to self-education, cross-contamination between trained and untrained endoscopists, and consultation with expert endoscopists, the untrained group may have been exposed to some level of training and could therefore have shown better outcomes than expected.

Given the blinded, retrospective analysis of T1 CRC recognition, extracted from the national pathology database, we were not able to calculate specificity. Information regarding the total number of benign lesions treated in the different centers cannot be retrieved due to lack of a national/regional registry.

The observed sensitivity in the preceding and final image-based practice cases of the e-learning program (82 % and 82 %, respectively) was higher than the sensitivity observed in real-life practice. High quality images and videos, unlimited assessment time, and optimal visualization of the area of interest could have contributed to this high performance. This contradicts real-life circumstances, where polyp cleaning, scope positioning, and time pressure may interfere with optimal assessment conditions. The dictated structured approach during the practice cases may also not have been applied during live endoscopies. A Hawthorne effect could also have contributed to better optical diagnosis in the e-learning program compared with real-life practice. While this part of the study was initially designed as a tool to measure the effect of optical training on recognition of T1 CRCs, this appeared not to be appropriate for estimation of the baseline sensitivity for T1 CRCs, given the fact that participating endoscopists studied beforehand and used study materials during the practice cases. Altogether, this suggests that endoscopists might be good at diagnosing T1 CRCs under the right circumstances, but these circumstances might not be reached during real-life clinical practice, resulting in a lower sensitivity for T1 CRCs. As endoscopists were unaware of the clinical part of our study, our data reflect real-life endoscopic practice for T1 CRCs at the community level.

Given the pragmatic nature of this study, with the aim of including as many Dutch centers as possible to evaluate the effects of training in optical diagnosis of T1 CRCs at the national level, no sample size calculation was performed. Post hoc sample size analysis showed a minimum inclusion of 154. Given the 528 inclusions in this study, it is assumed that we have more than enough power to support our findings.

In conclusion, in this prospective, multicenter, intervention study, it was shown that training in optical recognition of T1 CRCs was associated with an increase in sensitivity for recognition of T1 CRCs in clinical practice. Better recognition led to a higher rate of selecting an appropriate en bloc local excision technique, resulting in higher R0 resection rates. There is, however, still room for significant improvement, as recognition only resulted in the selection of a dedicated resection technique in 50 % of cases, and referral for primary surgery in 41 %. The focus, therefore, should be not only on recognition, but also on appropriate treatment. This may be an important step toward more organ-preserving strategies.

## Supplementary Material

Supplementary material Zusatzmaterial
