# Supplementary material for: Effect of optical diagnosis training on recognition and treatment of submucosal invasive colorectal cancer in community hospitals: a prospective multicenter intervention study
Source: Endoscopy. 2024 Jun 11;56(10):770–9. doi: 10.1055/a-2313-4996 (PMC11436291; doi:10.1055/a-2313-4996)

Supplementary material

Effect of optical diagnosis training on recognition and treatment of submucosal invasive colorectal cancer in community ho spitals: a prospective multicenter intervention studyl)

Authors: **Lonne W.T. Meulen**, Krijn J.C. Haasnoot, Marije Vlug, Frank H.J. Wolfhagen, Martine Baven-Pronk, Michael P.J.A. van der Voorn, Matthijs P. Schwartz, Lauran Vogelaar, Wouter de Vos tot Nederveen Cappel, Tom Seerden, Wouter Hazen, Ruud Schrauwen, Lorenza Alvarez-Herrero, Ramon-Michel Schreuder, Annick B. van Nunen, Esther Stoop, Gijs de Bruin, Philip Bos, Willem A. Marsman, Edith Kuiper, Marc de Bièvre, Yasser Alderlieste, Robert Roomer, John Groen, Marloes Bigirwamungu-Bargeman, Peter D. Siersema, Sjoerd G. Elias, Ad A.M. Masclee, Leon M.G. Moons (on behalf of the OPTICAL-STAR study team and the Dutch T1 CRC Working Group)

TABLE OF CONTENTS:

Supplementary material Part 1s: Details of e-module for OPTICAL training

A detailed description of e-module 2

Table 1s Questions and answer options asked in pre- and post-test 4

Fig. 1s a,b Examples of e-learning in online web-based module 5

Fig. 2s E-module participation 6

Supplementary material Part 2s: Clinical cohort

Table 2s Baseline characteristics of trained and untrained endoscopists 7

Fig. 3s Directed acyclic graph T1 CRC recognition 8

Fig. 4s a–d Variability between centers regarding optical diagnosis and treatment outcomes of T1 CRCs 9

Fig. 5s a–d Distribution between centers regarding optical diagnosis and treatment outcomes of T1 CRCs 11

Supplementary material

Supplementary material Part 1s: Details of e-learning for OPTICAL training

Before starting the e-learning participants provided consent for the usage of their results for this study and filled in a baseline questionnaire about their experience as an endoscopist, supplemented with information about the endoscopists provided by the principal investigators of each center. Progression through the e-learning was logged, and only after completion, participants could proceed to the post-test, scheduled 4-6 weeks after the e-learning.

Practice cases

The practice cases consisted of 40 real-time cases of LNPCPs selected from a large prospective database of registered LNPCPs in a tertiary hospital (University Medical Center Utrecht). Polyps contained either low-grade dysplasia (LGD; n=19), high-grade dysplasia (HGD; n=11), or T1 CRC (n=10) as determined by the golden standard; pathology reports. All cases containing T1 CRC were revised to determine the level of submucosal invasiveness; deep invasion was defined as invasion depth ≥1000 μm or Kikuchi SM2-3. Three cases were classified as superficial T1 CRC and seven cases as deep invasive T1 CRC. Cases in the preceding and final practice set were equal but arranged in different order. None of these cases were used in the e-learning itself.

For each case, we provided multiple images and/or video material of the whole polyps in both white-light and advanced imaging (narrow-band imaging [NBI]). We selected cases on which all features of the OPTICAL-model could be assessed. Size and location were provided in a textbox. Also, if spontaneous bleeding was unlikely to be adequately assessed on the imagery, this feature was stated in the text box. For each case, participants were asked ten questions, for which they had to give a predefined answer (Table S1). First, seven questions concerning the characterization of the polyp by features associated with a risk of invasive cancer were asked. Second, participants had to predict their optical diagnosis (LGD, HGD, superficial T1 CRC, or deep-invasive T1 CRC) and give their recommended therapy: piecemeal resection, en-bloc resection, or surgical resection. Finally, they had to provide the risk of invasive carcinoma in the assessed polyp (0-100%).

Supplementary material

For the golden standard of the polyp features, we used the answers provided by previously trained endoscopists (participants in the original OPTICAL study) to reach a consensus. Questions reaching <70% consensus were reviewed and the final decision for the answer key was made by an expert endoscopist and developer of the e-learning (LMM). The risk of carcinoma was calculated by using the OPTICAL-model with the features of the answer key as input. (Figure S1)

To evaluate the lasting effect of the e-learning, participants were asked to wait 4-6 weeks after finishing the e-learning before proceeding to the finale practice cases.

Design of e-learning

The e-learning was designed to explain the necessity of optical diagnosis in LNPCPs and to explain in detail all aspects of the OPTICAL model. (Figure S3) It consisted of three chapters and six subchapters: I) Why is optical diagnosis important for your practice, II) The making of the OPTICAL model, and III) Features of the OPTICAL model. This last chapter was subdivided into a) Stepwise approach for optical diagnosis, b) Surface morphology, c) White light features of malignancy: Depression & Spontaneous bleeding, d) Advanced Imaging: Narrow-Band Imaging, e) Advanced Imaging: Pit pattern analysis and f) Combined approach of all features: Using the Optical model. All chapters were video lectures interrupted with interactive case-based questions. The total duration of the e-learning was approximately 3 hours.

Supplementary material

Table 1s Questions and answer options asked in practice cases

Question	Option 1	Option 2	Option 3	Option 4	Option 5
Question 1 Surface morphology	Homogenous granular	Granular with large nodule > 10mm	Granular with non-erythematous area	Non-granular	-
Question 2 Depression	Yes, well demarcated	Yes, not well demarcated	No depression	-	
Question 3 Spontaneous Bleeding	Yes	No	Cannot be assessed	-	
Question 4 Vessel distribution	Regular	Irregular	Absent	-	
Question 5 Vessel diameter	Even	Uneven	Absent	-	
Question 6 Hiroshima	A	B	C1	C2	C3
Question 7 NICE	NICE 1	NICE 2	NICE 3	-	
Question 8 Optical diagnosis	LGD	HGD	T1 superficial	T1 deep	-
Question 9 Therapy	Endoscopic resection (piecemeal)	En-bloc resection	Surgery	-	
Question 10 OPTICAL %	% (0-100)	-			

Supplementary material

Fig. 1s a,b Examples of e-learning in an online web-based module

a

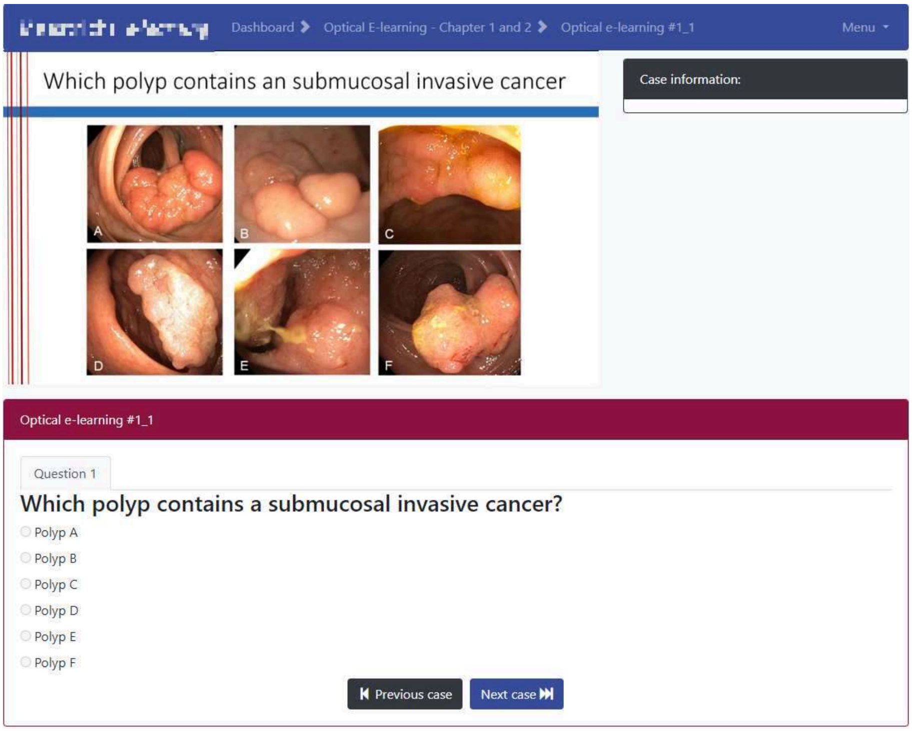

b

Supplementary material

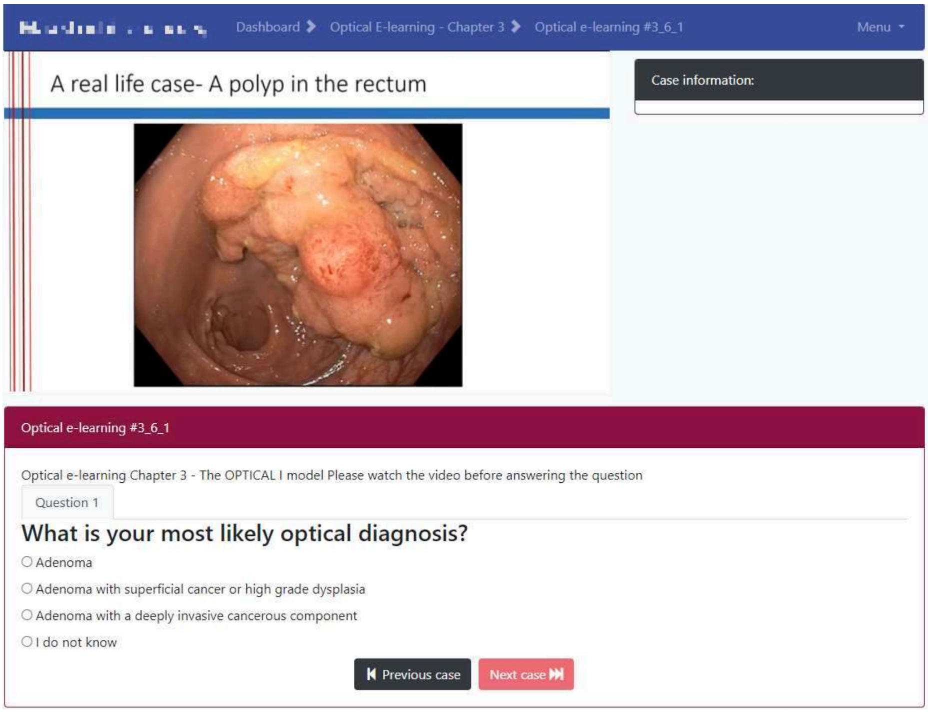

Supplementary material

Fig. 2s E-module participation

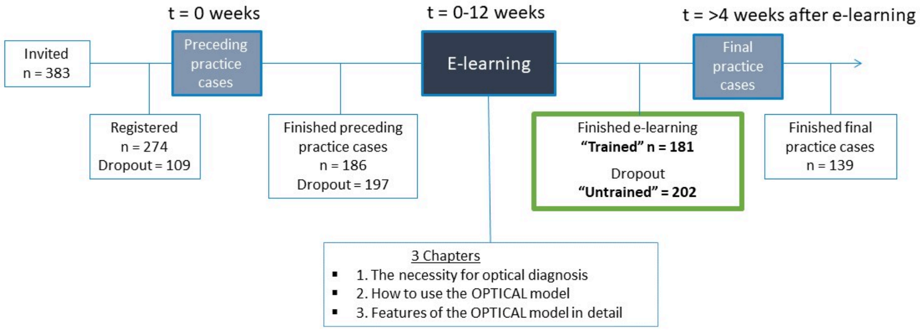

Supplementary material

Supplementary material Part 2s: Clinical cohort

Table 2s Baseline characteristics of trained and untrained endoscopists

	Overall N=251	Trained N=118	Untrained N=133	P-value
Endoscopy experience in years, median (IQR)	10 (6-16)	9 (6-15)	12 (6-19)	0.120
Focus area, n (%)				*0.003*
- Colorectal	39 (16%)	31 (27%)	8 (6%)	
- Liver	19 (8%)	8 (7%)	11 (8%)	
- HPB	23 (9%)	12 (10%)	11 (8%)	
- Esophagus/Stomach	7 (3%)	3 (3%)	4 (3%)	
- Functional	7 (3%)	3 (3%)	4 (3%)	
- IBD	41 (16%)	14 (12%)	27 (20%)	
- General	49 (20%)	22 (19%)	26 (20%)	
- Oncology	2 (1%)	1 (1%)	1 (1%)	
- Unknown	64 (26%)	22 (19%)	41 (31%)	
Screening program endoscopist, n (%)	160 (64%)	89 (77%)	70 (53%)	*<0.001*
Uses (virtual) chromoendoscopy, n (%)	152 (61%)	88 (76%)	64 (47%)	*<0.001*
Performs EMR ≥20mm, n (%)	90 (36%)	57 (49%)	32 (24%)	*<0.001*
Frequently using advanced endoscopy techniques, n (%)	34 (14%)	28 (24%)	6 (5%)	*<0.001*
Dedicated in recognition of T1 CRCs, n (%)	104 (41%)	62 (53%)	42 (32%)	*<0.001*
Total colonoscopies performed, median (IQR)	3500 (2112-5570)	3478 (2463-5000)	3600 (1600-6000)	0.757
Frequently consulting expert, n (%)	184 (73%)	92 (79%)	91 (68%)	*0.002*

Fig. 3s Directed acyclic graph T1 CRC recognition

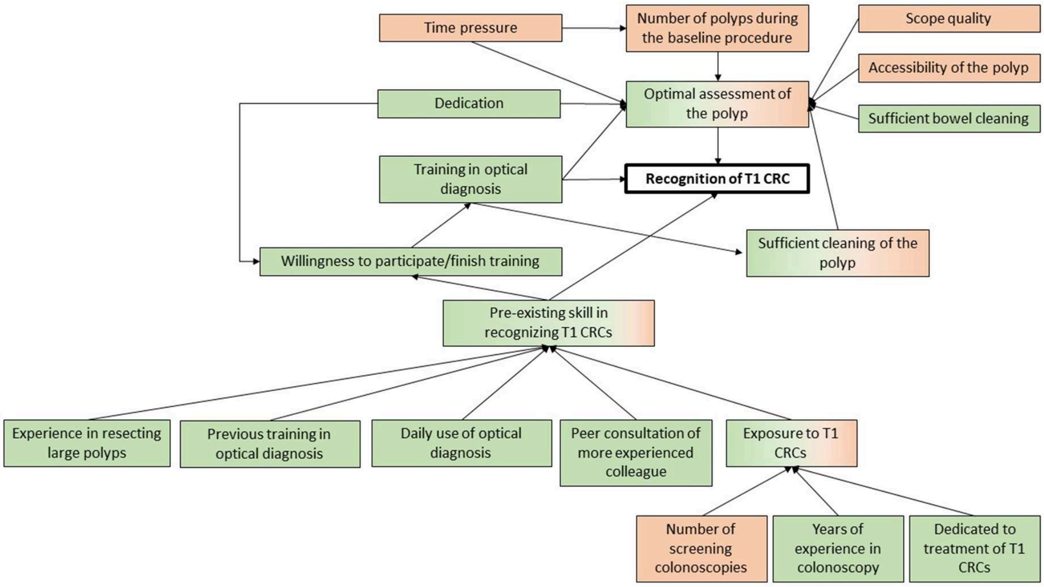

Green: data available in cohort

Orange: data not available in cohort

Supplementary material

Fig. 4s a–d Variability between centers regarding optical diagnosis and treatment outcomes of T1 CRCs.

a

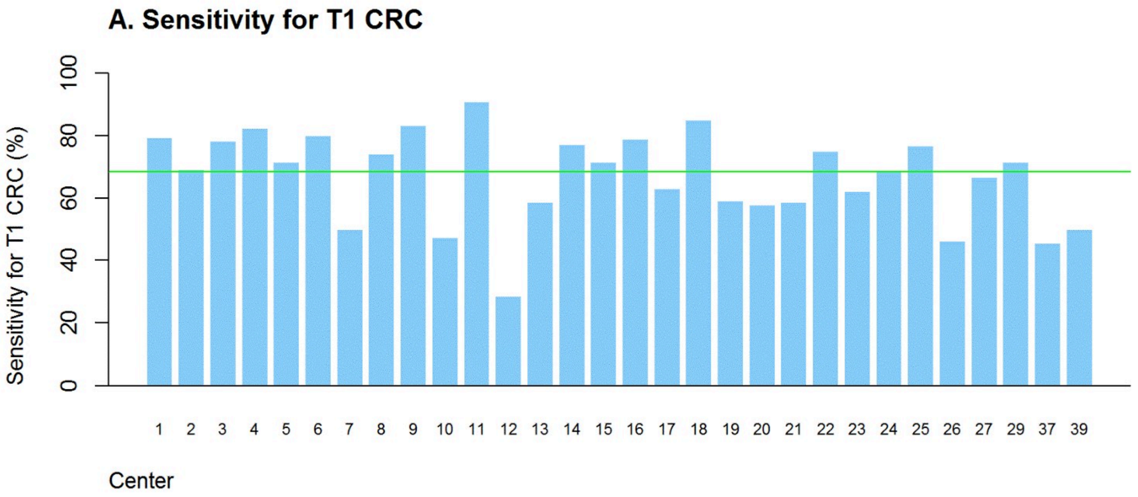

b

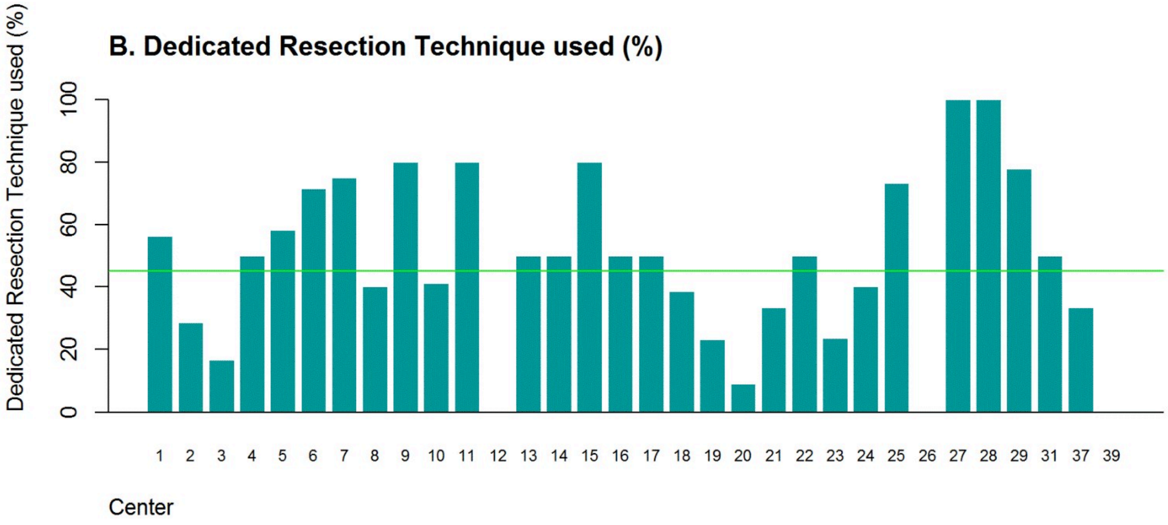

Supplementary material

c

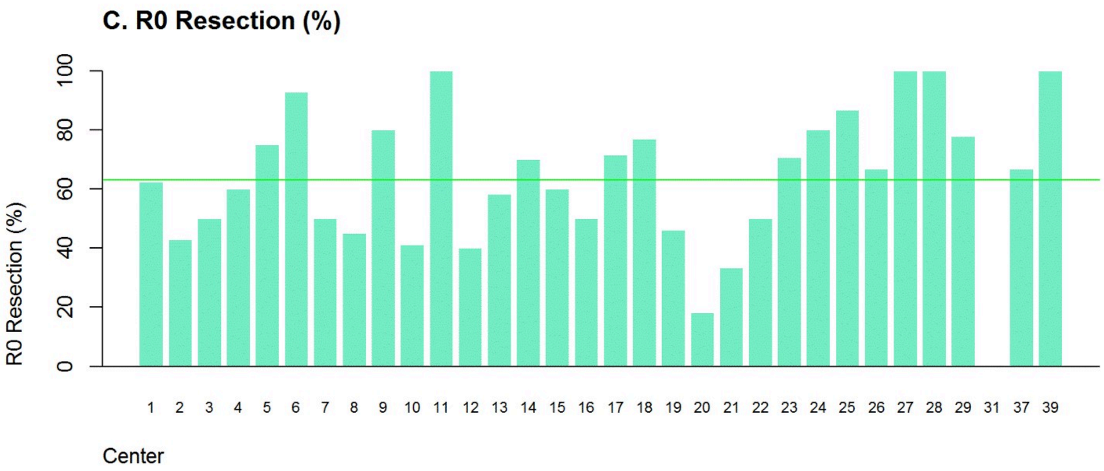

d

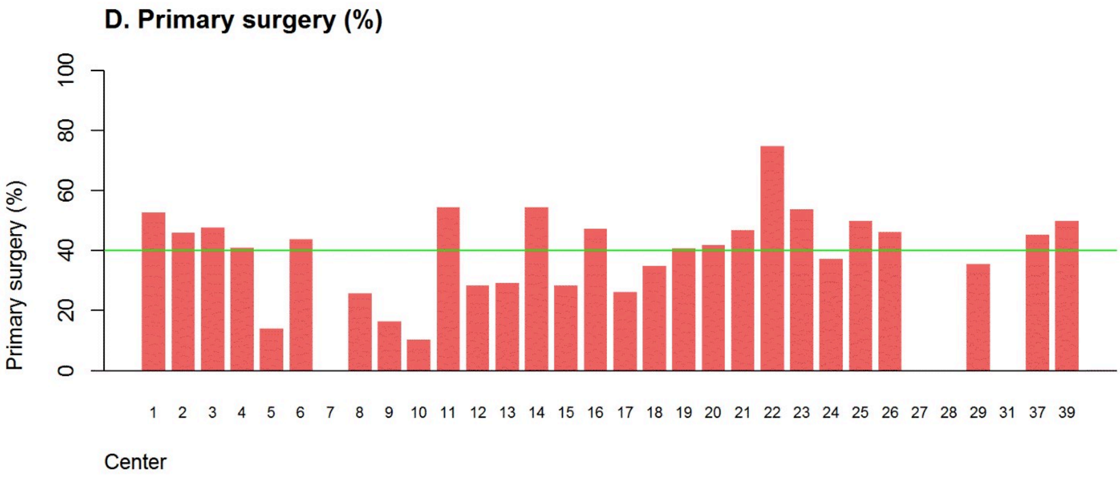

Supplementary material

Fig. 5s a–d Distribution between centers regarding optical diagnosis and treatment outcomes of T1 CRCs.

a

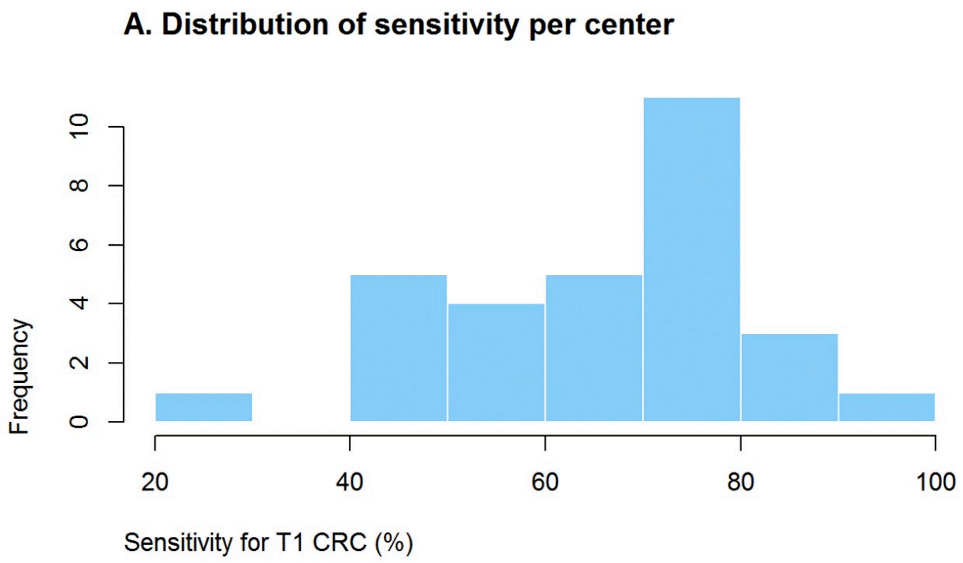

b

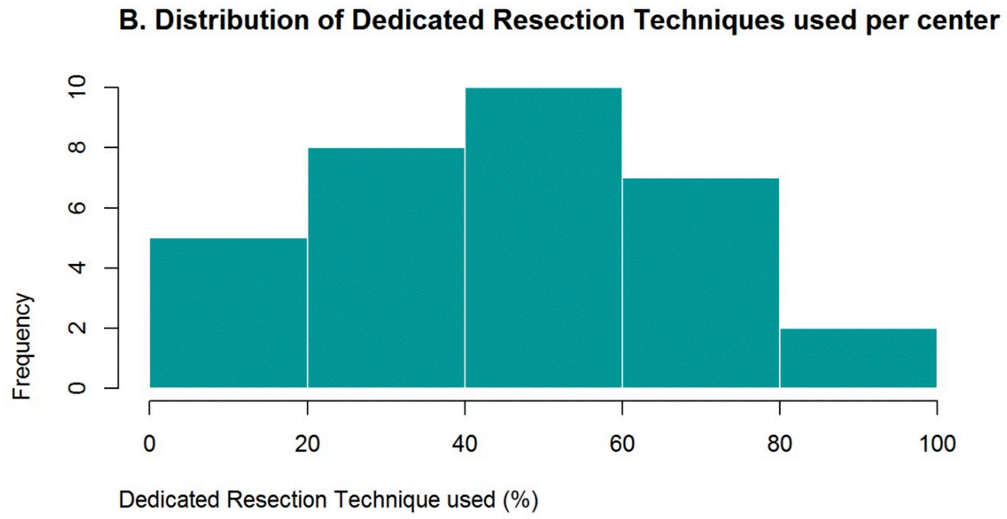

Supplementary material

c

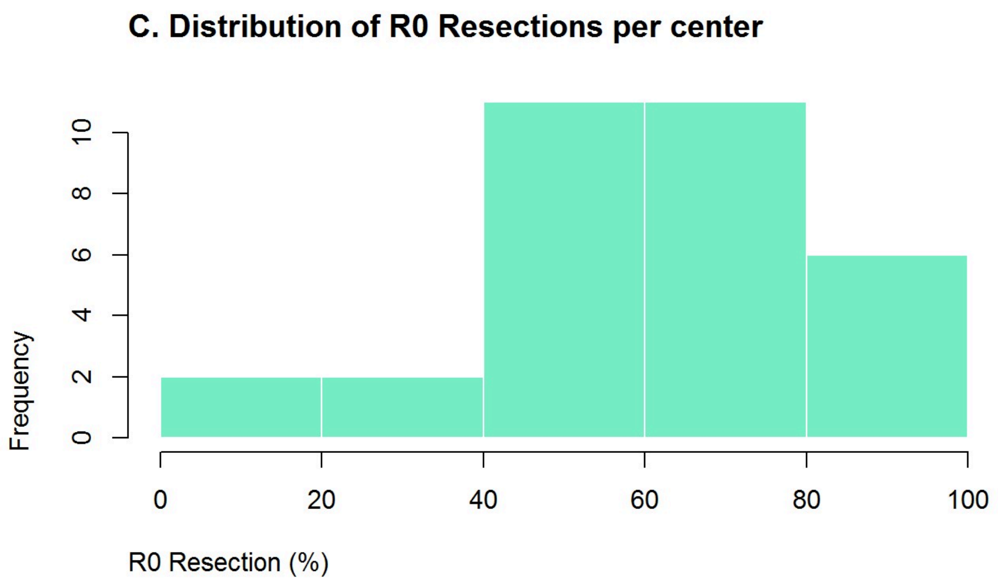

d

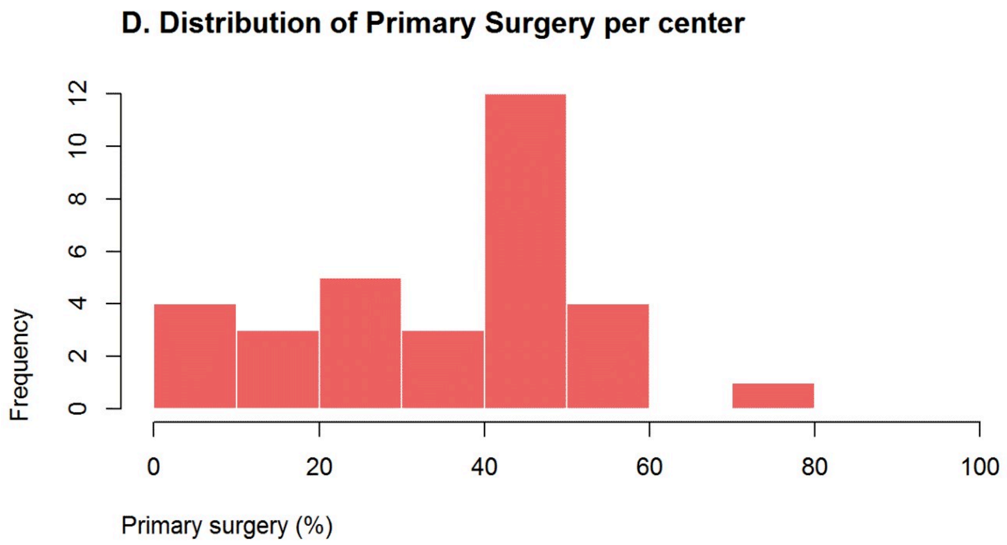

Supplement: Supplementary file 1 — Supplementary material [file 22958supmat_10-1055-a-2313-4996.pdf]
